# Carbapenem-resistant *Pseudomonas aeruginosa*: an assessment of frequency of isolation from ICU versus non-ICU, phenotypic and genotypic profiles in a multinational population of hospitalized patients

**DOI:** 10.1186/s13756-022-01187-8

**Published:** 2022-11-30

**Authors:** Christian M. Gill, David P. Nicolau, on behalf of the ERACE-PA Global Study Group

**Affiliations:** 1 Center for Anti-Infective Research & Development Hartford Hospital, 80 Seymour Street, Hartford, CT 06102 USA; 2 Division of Infectious Diseases, Hartford Hospital, Hartford, CT USA

**Keywords:** *Pseudomonas aeruginosa*, Carbapenem-resistant, Ceftolozane/tazobactam, Ceftazidime/avibactam, Nosocomial pneumonia

## Abstract

**Background:**

Historically, multi-drug resistant organisms have been associated with the ICU setting. The present study sought to define the frequency of isolation from ICU versus non-ICU, phenotypic and genotypic profiles of carbapenem-resistant *Pseudomonas aeruginosa* (CR-PA) from a global cohort.

**Methods:**

Multicenter surveillance study (17 centers from 12 countries) including 672 CR-PA isolates from 2019 to 2021. Phenotypic carbapenemase testing was assessed. Genotypic carbapenemase testing was conducted (CarbaR and CarbaR NxG) to detect β-lactamases. Broth microdilution MICs were established for ceftazidime, cefepime, ceftolozane/tazobactam, and ceftazidime/avibactam.

**Results:**

59% of CR-PA were isolated from patients outside the ICU. The most common source in ICU and non-ICU patients was respiratory (55% and 30%, respectively). In the ICU, 35% of isolates were phenotypically carbapenemase-positive versus 29% for non-ICU. VIM was the most common carbapenemase (54% and 44%, respectively) followed by GES (27% and 28%, respectively). Susceptibility to ceftazidime or cefepime were relatively low in ICU (39% and 41% of isolates, respectively) and non-ICU (47% and 52% of isolates, respectively). Ceftolozane/tazobactam and ceftazidime/avibactam were more active with 56% and 66% of isolates susceptible in the ICU while 65% and 76% in non-ICU, respectively. When carbapenemase-negative, 86% and 88% of ICU isolates were susceptible to ceftolozane/tazobactam and ceftazidime/avibactam. Similarly, in the carbapenemase-negative, non-ICU isolates 88% and 92% of isolates were susceptible, respectively.

**Conclusion:**

Although multidrug resistant pathogens are often regarded as a challenge in the ICU population, the majority of CR-PA were isolated from non-ICU patients. Implementing phenotypic/genotypic testing will assist in guiding treatment. Carbapenem-resistance in *P. aeruginosa* should be regarded as a surrogate for MDR and this phenotype is increasingly prevalent outside the ICU.

## Background

Carbapenem-resistant *P. aeruginosa* (CR-PA) challenges clinicians due to the limited available treatments. An assessment of patient-related risk factors are needed to determine which patients are most likely to be infected with CR-PA to select empiric therapy in the setting of infection. ICU status has been associated with CR-PA infections, although; numerous other factors such as previous exposure to carbapenems, transfer from a skilled nursing facility among, and microbiologic history are also important considerations [1]. Previous data from US medical centers highlighted the increasing prevalence of CR-PA in the non-ICU patients [2]. Thus, the present study sought to evaluate the prevalence of carbapenem-resistant *P. aeruginosa* in the ICU and non-ICU settings from a global surveillance program. Phenotypic and genotypic carbapenemase detection was assessed in each cohort to consider how this information can inform therapeutic decisions.

## Methods

### Isolates and minimum inhibitory concentration determination

Isolates were collected during the ERACE-PA Global Surveillance program [3]. Submitting sites identified clinically relevant CR-PA per local standards. Non-duplicate isolates from any body source or specimen type were sent if they were determined to be resistant to any carbapenem (meropenem or imipenem) using conventional susceptibility testing methods. Seventeen sites in 12 countries including the United States, Germany, Brazil, Turkey, Israel, Spain, Kuwait, South Africa, Colombia, Greece, Saudi Arabia, and Italy were included (Table 1). Source of culture and patient location (ICU versus non-ICU) were submitted as available. The present study assessed 672 CR-PA isolates from the program where location (ICU versus non-ICU) status was available.

**Table 1:** Percent of CR-PA isolates in ICU and non-ICU by geographic region

Geographic region	Percent of CR-PA from ICU patients (n = 275) (%)	Percent of CR-PA from non-ICU patients (n = 397) (%)
Europe	39	37
Middle East	22	25
South America	8	10
United States	17	23
Africa	14	5

Broth microdilution MICs were determined and interpreted per CLSI standards for ceftazidime, cefepime, ceftazidime/avibactam, and ceftolozane/tazobactam [4]. The proportion of isolates susceptible to each agent is described and the 95% confidence interval for the estimate was calculated per CLSI standards.

### Phenotypic and genotypic resistance determinants

Isolates underwent phenotypic carbapenemase testing using the modified carbapenem inactivation method (mCIM) as previously described [3, 4]. Isolates positive for carbapenemase production were assessed using the CarbaR or the research-use-only CarbaR NxG to assess for genotypic carbapenemases (VIM, NDM, IMP, KPC, OXA-48, GES) [3]. Additionally, phenotypically carbapenemase-negative isolates that were ceftolozane/tazobactam-non-susceptible underwent CarbaR or CarbaR NxG testing.

To assess the potential impact of carbapenemase-testing implementation on the ability to select active therapy while awaiting susceptibility testing of the novel-β-lactam- β-lactamase inhibitor combinations, the percent of isolates susceptible to ceftolozane/tazobactam and ceftazidime/avibactam by phenotypic and genotypic carbapenemase status and the 95% confidence interval of the estimate were assessed. Groups of isolates that the estimate or the 95% confidence interval cross 90% susceptibility for an agent were considered ideal [5].

## Results

Two hundred seventy-five (41%) isolates were cultured from ICU patients compared with 397 (59%) from non-ICU settings. The most common source in both cohorts was respiratory (55% and 30%, respectively). In ICU patients, blood was the second most common source (13%) followed by urine (10%) and intra-abdominal (1%) while 21% were other or non-specified. In non-ICU patients, urine was the second most common source (28%) followed by blood and intra-abdominal (9% and 2%, respectively) while 31% were other or non-specified.

**Fig. 1:**
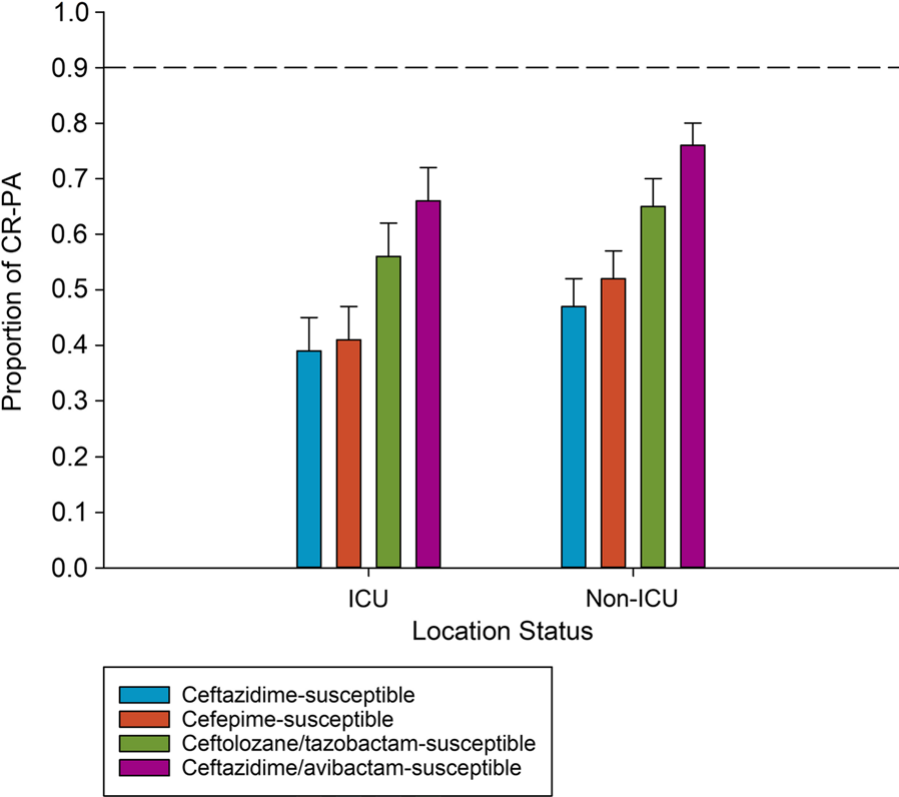
In vitro potency of anti-pseudomonal cephalosporins against carbapenem-resistant *P. aeruginosa* (CR-PA) from ICU and non-ICU settings in a global surveillance program. ICU: n = 275; MIC50/90 (mg/L) for Ceftazidime (32/>64), cefepime (16/>64), ceftolozane/tazobactam (2/>64), ceftazidime/avibactam (4/64). Non-ICU: n = 397; MIC50/90 (mg/L) for Ceftazidime (16/>64), cefepime (8/>64), ceftolozane/tazobactam (1/>64), ceftazidime/avibactam (4/64)

Susceptibility profiles of ICU and non-ICU isolates were similar, although; numerically lower percent susceptible in the ICU isolates across each test agent (Fig. 1). Overall, cross-resistance was high among the CR-PA assessed regardless of ICU or non-ICU status. Isolates remained susceptible to ceftazidime in 39% and 47% of isolates from the ICU or non-ICU, respectively. Similarly, cefepime was active against 41% and 52% of isolates in each setting, respectively. In contrast, ceftolozane/tazobactam and ceftazidime/avibactam retained in vitro activity against 56% and 66% of isolates in the ICU, respectively. A similar trend was seen in the non-ICU with 65% and 76% of isolates testing susceptible to each agent, respectively.

**Fig. 2:**
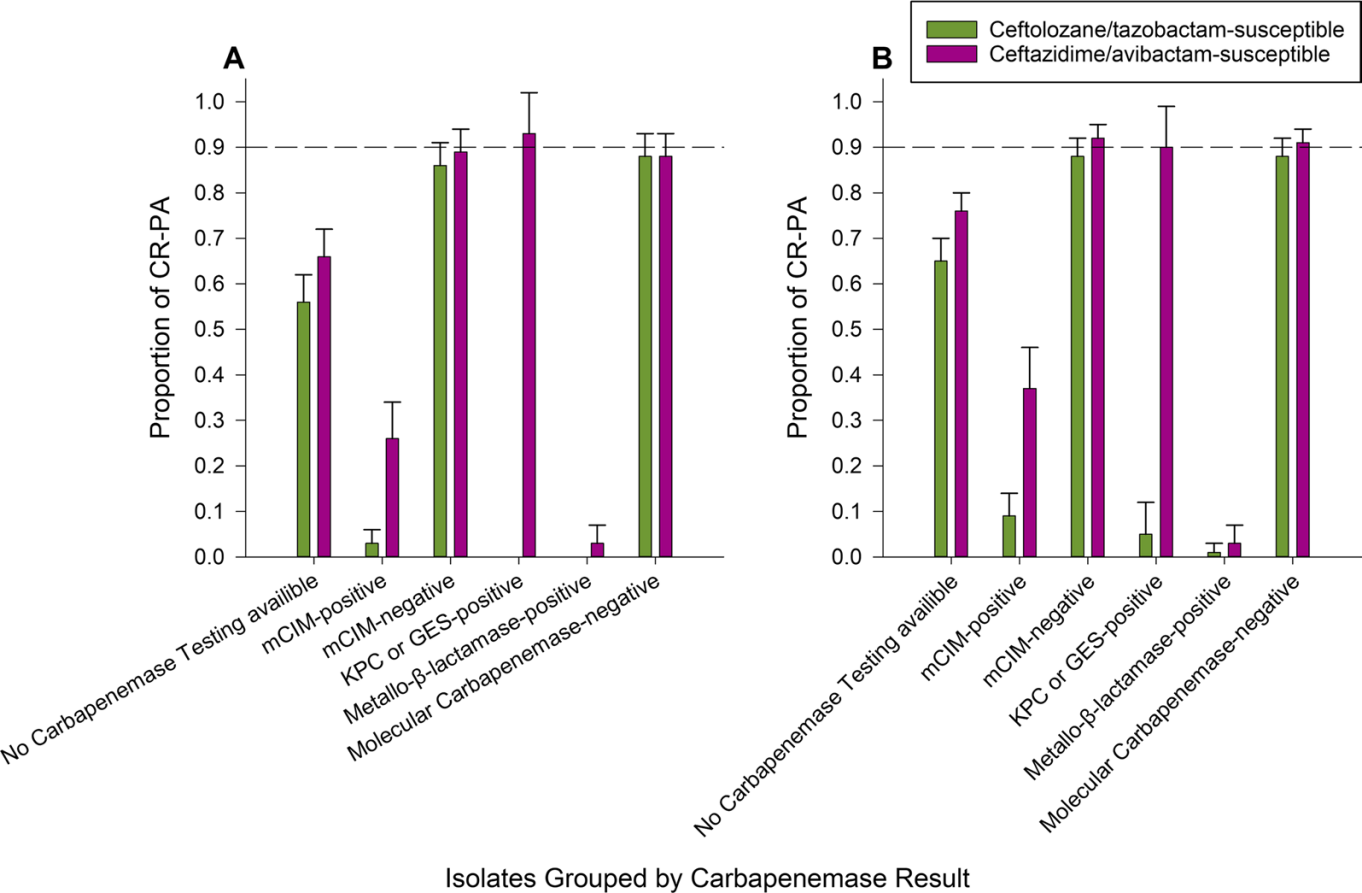
Ceftolozane/tazobactam and ceftazidime/avibactam susceptibility results of subgroups of isolates by phenotypic or genotypic carbapenemase testing results from patients in the ICU (**A**) or non-ICU (**B**). These data can guide clinicians to rational antimicrobial selection while awaiting susceptibility testing results for ceftolozane/tazobactam and/or ceftazidime/avibactam. Ruling out carbapenemase can inform clinicians that either ceftolozane/tazobactam or ceftazidime/avibactam is likely active and thus be rational selections. Conversely, non-specific detection phenotypically of a carbapenemase is associated with poor susceptibility to either agent suggesting alternative therapies. Molecular detection of specific carbapenemase classes can further refine agent selection

A similar percentage of each cohort was phenotypically carbapenemase positive (ICU 35%; non-ICU 29%). VIM was the most commonly encountered genotypic carbapenemase among carbapenemase positive isolates (ICU 54%; non-ICU 44%). GES was the second most common amongst carbapenemase positive organisms in both cohorts with 27% and 28%, respectively. The remaining ICU isolates positive for a carbapenemase by PCR included IMP (7%), NDM (5%), VIM + KPC (4%), VIM + IMP (3%), KPC (< 1%) and VIM + OXA-48 (< 1%). In non-ICU isolates, remaining PCR positive isolates included NDM (7%), IMP (6%), KPC (6%), VIM + KPC (4%). Interestingly, nine ICU isolates and six non-ICU isolates were mCIM negative but harbored GES-β-lactamases. Conversely, seven ICU isolates and 12 non-ICU isolates tested phenotypically positive on mCIM but lacked tested carbapenemase targets. Figure 2 provides the percent of isolates susceptible to ceftolozane/tazobactam, and ceftazidime/avibactam based on phenotypic or genotypic carbapenemase data to assess the likelihood each agent is susceptible if CR-PA is encountered while awaiting susceptibility testing. mCIM negative isolates were highly likely to be susceptible to ceftolozane/tazobactam and ceftazidime/avibactam with 86% and 89% of isolates in the ICU testing susceptible to each agent, respectively. A similar trend was noted in non-ICU patients with 88% and 92% testing susceptible, respectively. Phenotypic detection positive for carbapenemases can alert clinicians alternative therapies are need as ceftazidime/avibactam and ceftolozane/tazobactam had relatively low susceptibility (Fig. 2). Conversely, detection of specific carbapenemase such as KPC or GES was associated with high in vitro activity for ceftazidime/avibactam with 93% and 90% of isolates testing as susceptible in the ICU and non-ICU, respectively. Detection of any metallo-β-lactamase, as expected, resulted in low in vitro activity against both cephalosporin-β-lactamase combinations assessed.

## Discussion

The present study is consistent with previously published data where CR-PA is a growing issue in both the ICU and non-ICU settings. Indeed, 59% of the CR-PA in the present study were isolated from patients in non-ICU settings consistent with the findings that CR-PA is not only an ICU based pathogen [2]. Similarly, respiratory tract was the most common culture source in both the ICU and non-ICU patients. These data provide significant insights for therapeutic options for hospital acquired pneumonia (HAP) including hospital acquired pneumonia requiring ventilation (V-HAP) and HAP not requiring ventilation (NV-HAP) for the non-ICU isolates as these patients may undergo transitions from the ward to the ICU for escalated care. Data from ICU patients may provide insights into the susceptibility pattern of CR-PA responsible for ventilator associated pneumonia (VAP) [6].

V-HAP represents a challenging clinical syndrome where mortality is estimated at 28%, notably higher than VAP (18%) and NV-HAP (15%) [6]. The mechanisms behind increased mortality in V-HAP compared with the syndromes are unclear, however; inappropriate empiric therapy due to higher prevalence of resistant organisms has been associated with increased complications [7]. Cefepime represents first line therapy for HAP/VAP [5]. However, regardless of ICU or non-ICU status in the present study, cefepime activity was markedly worse than ceftolozane/tazobactam or ceftazidime/avibactam in the setting of CR-PA. This is echoed in a retrospective cohort study where infection with CR-PA that were susceptible to other typical β-lactam agents (i.e., cefepime, ceftazidime, and piperacillin/tazobactam) was associated with higher 30-day mortality than infection with CR-PA resistant to these agents [8]. Although treatment regimens were not assessed, a potential explanation for this finding is the use of non-pharmacodynamically optimized dosing regimens in the setting of higher MICs to the susceptible β-lactams due to cross-resistance. We previously found that the elevated MICs to cefepime and ceftazidime in CR-PA, although susceptible per interpretive criteria, necessitate pharmacodynamically optimized doses to achieve PKPD targets [9]. Further investigation is needed to evaluate if pharmacodynamically optimized dosing can improve outcomes for CR-PA susceptible to typical β-lactams. When treating CR-PA, clinicians should consider more potent alternatives (e.g., ceftolozane/tazobactam or ceftazidime/avibactam) until such data are available.

Cross-resistance amongst *P. aeruginosa* must also be considered during transitions of care from non-ICU to ICU settings which may be prominent in V-HAP [5, 6]. Meropenem is often considered an escalation in this setting of cefepime failure. The present cohort exclusively comprised of carbapenem-resistant isolates however Lob and colleagues reported in a US cohort of *P. aeruginosa* that cefepime-non-susceptibility was accompanied with meropenem-susceptibly in only 36% and 40% of isolates in the ICU and non-ICU compared with 77% and 84% of isolates for ceftolozane/tazobactam in each setting, respectively [10]. Similar to our findings, the same authors found meropenem-non-susceptibility was associated with 46% and 49% susceptibility to cefepime in the ICU and non-ICU, respectively. These data reinforce that *P. aeruginosa* with reduced susceptibility to cefepime and/or carbapenems may carry cross-resistance to the other agent. Contemporary antibiograms do not account for the fact that resistance to one β-lactam agent may be accompanied with elevated MICs to another. Other interventions (e.g., MDR- antibiograms or patient risk based algorithms) to stratify patients who would benefit from empiric escalation to more potent agents (e.g., ceftolozane/tazobactam and ceftazidime/avibactam) are warranted.

**Fig. 3:**
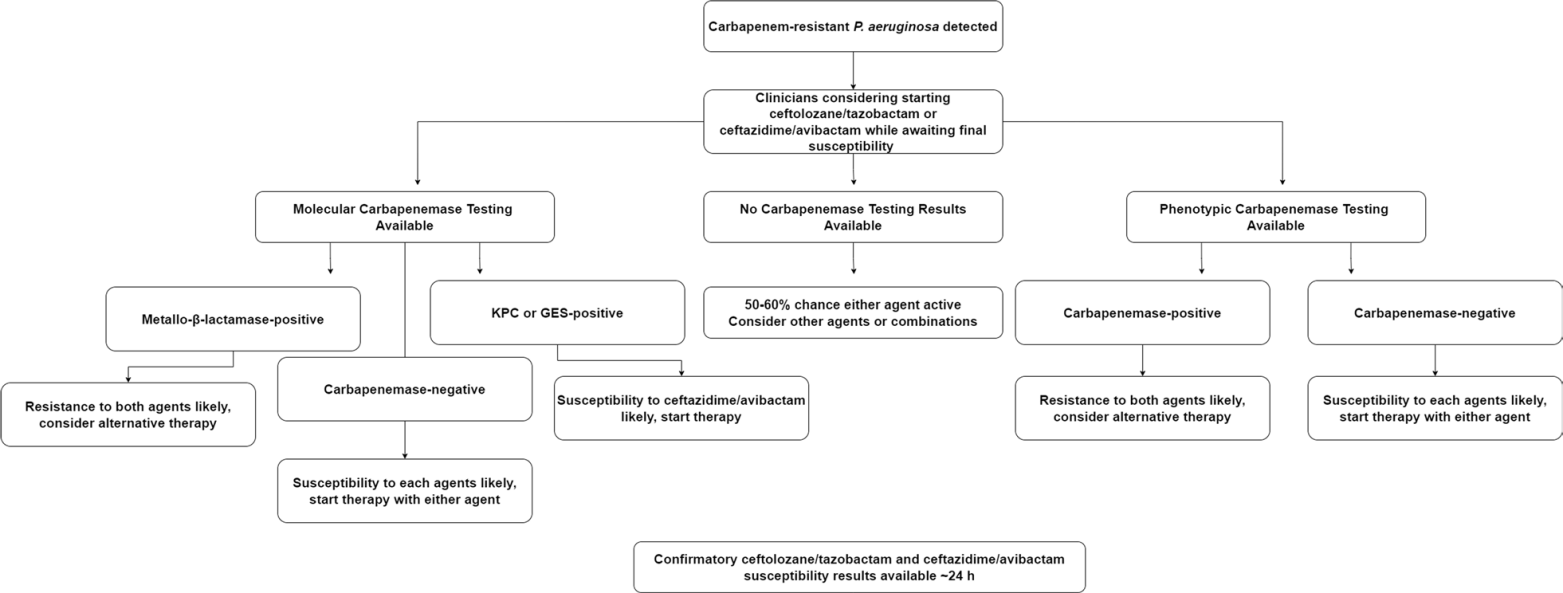
Example therapeutic pathway to determine antimicrobial selection based on phenotypic or genotypic carbapenemase testing while awaiting cascade susceptibility testing results for novel agents (e.g., ceftolozane/tazobactam or ceftazidime/avibactam) based on data in Fig. 2. Implementation can guide selection in the ICU and non-ICU setting

Cascade reporting of susceptibility results have been a standard practice for antimicrobial stewardship programs. In this model, expanded agents (e.g., ceftolozane/tazobactam and ceftazidime/avibactam) are tested when specific criteria are met (e.g., carbapenem-non-susceptibility). Of note, novel agents may not be immediately available on automated susceptibility testing platforms and clinical laboratories must utilize other methods for testing such as gradient diffusion strips or disk diffusion which may take another 24 h for results [11]. A quasi-experimental study in a regional health system found that the median time from known meropenem-non-susceptibility to ceftolozane/tazobactam testing results was 25.4 h [12]. Based on the present study, knowing the carbapenemase status via phenotypic or genotypic methods may increase the reliability to appropriately select antimicrobials in the setting of CR-PA while awaiting confirmatory susceptibility testing. Indeed, the mCIM was used in this study which requires 18–24 h to obtain a result, which is likely similar to the timeline for confirmatory susceptibility testing [4, 11, 13]. However, more rapid methods (i.e., CarbaNP, immunoassays) or molecular diagnostics (i.e., the CarbaR) are available in as little as 30 min which would provide actionable data sooner [4, 10, 13]. Figure 3 describes a potential therapeutic decision making algorithm in response to carbapenemase testing as described in Fig. 2. Indeed, ruling out a carbapenemase (phenotypically or genotypically) can provide clinicians confidence in starting ceftolozane/tazobactam or ceftazidime/avibactam therapy while awaiting confirmatory susceptibility testing. Similarly, Molecular detection of KPC or GES can alert clinicians to select ceftazidime/avibactam as it is more likely to be active. Finally, detection of metallo-beta-lactamases results in poor activity of either ceftolozane/tazobactam or ceftazidime/avibactam alerting clinicians to use alternative agents or combinations. Our data shows such carbapenemase status therapy stratification can be useful in both the ICU and non-ICU setting.

Interestingly, phenotypic and genotypic mis-matches were detected in our cohorts (i.e., mCIM-negative, GES-positive). Indeed, not all GES-subtypes have been considered carbapenemases some are considered ESBLs [14, 15]. Although PCR-testing used cannot differentiate between alleles previously categorized as ESBLs or carbapenemases, detection of such enzymes can still guide therapeutic decisions as ceftazidime/avibactam is more likely to be active than ceftolozane/tazobactam regardless of variant (Fig. 2) [3]. More data are needed to dictate optimal therapy when GES-harboring *P. aeruginosa* are detected.

## Conclusion

In conclusion, in a global cohort of CR-PA, a significant portion of isolates were obtained from patients in non-ICU settings and the respiratory tract was the most common source for both ICU and non-ICU patients. Cross-resistance to cefepime and ceftazidime were common, although; ceftolozane/tazobactam and ceftazidime/avibactam maintained activity in more isolates regardless of ICU or non-ICU status. Using these data from the ICU may be informative for VAP treatment guidelines. Similarly, the high proportion of CR-PA obtained from non-ICU patients must be considered for escalation of therapy during transitions of care from the ward to the ICU in the setting of HAP and most notably, V-HAP.

## Data Availability

The datasets used and/or analyzed during the current study are available from the corresponding author on reasonable request.

## Abbreviations

CR-PA: Carbapenem-resistant *Pseudomonas aeruginosa*
MIC: Minimum inhibitory concentration
VIM: Verona integron-encoded metallo-β-lactamase
NDM: New Delhi metallo-β-lactamase
IMP: Imipenemase
KPC: Klebsiella pneumoniae carbapenemase
OXA-48: Oxacillinase-48
GES: Guiana-extended-spectrum β-lactamase
mCIM: Modified carbapenem inactivation method
VAP: Ventilator acquired pneumoniae
V-HAP: Ventilated hospital associated pneumonia
NV-HAP: Non-ventilated hospital acquired pneumonia
PKPD: Pharmacokinetics/pharmacodynamics
MDR: Multi-drug resistance
